# Safety of cholecystectomy in patients under antithrombotic Drugs: A systematic review and meta-analysis

**DOI:** 10.12669/pjms.38.8.7032

**Published:** 2022

**Authors:** Xiaolan Shao, Xiuhong Cui

**Affiliations:** 1 Xiaolan Shao, Department of Operating Room, Dongtai people’s Hospital 2 Kangfu West Road, Dongtai 224200, Jiangsu Province, P.R. China; 2 Xiuhong Cui, Department of Operating Room, Dongtai people’s Hospital 2 Kangfu West Road, Dongtai 224200, Jiangsu Province, P.R. China

**Keywords:** Anticoagulants, Antiplatelets, Aspirin, Clopidogrel, Warfarin, Gastrointestinal surgery, Bleeding

## Abstract

**Objective:**

This review aimed to assess evidence on the safety of cholecystectomy in patients under antithrombotic therapy.

**Methods:**

PubMed, Embase, Science Direct, CENTRAL, and Google Scholar databases were searched from inception up to 20^th^ January 2022 for studies comparing outcomes of patients undergoing cholecystectomy surgeries with or without concomitant antithrombotic therapy.

**Results:**

Nine studies were included. Meta-analysis revealed that the use of antithrombotic medications had no statistically significant effect on intra-operative blood loss in patients undergoing cholecystectomy (MD: 82.31 95% CI: -283.38, 448 I^2^=98% p=0.66). However, incidence of blood transfusion (OR: 5.65 95% CI: 1.10, 28.86 I^2^=83% p=0.04) and bleeding complications (OR: 8.02 95% CI: 1.71, 37.58 I^2^ = 71% p=0.008) were significantly increased in patients under antithrombotic therapy. Pooled analysis indicated that cholecystectomy patients under antithrombotic are at an increased risk of conversion to open surgery (OR: 2.02 95% CI: 1.21, 3.36 I^2^=0% p=0.007). Meta-analysis revealed significantly shorter LOS in patients under antithrombotic (MD: -5.01 95% CI: -8.29, -1.73 I^2^=97% p=0.03).

**Conclusion:**

Current evidence from a limited number of studies indicates that the use of antithrombotic may be associated with an increased risk of bleeding-related complications in patients undergoing cholecystectomies. Antithrombotic use may also increase the risk of conversion to open surgery in patients undergoing laparoscopic cholecystectomies.

## INTRODUCTION

Minimizing patient complications and enhancing satisfaction are important goals of modern day healthcare practice. Indeed, along with surgeons, nursing personnel and other paramedical staff also have an important role in perioperative patient management.

Presence of comorbidities and associated medications can alter the perioperative management protocol of any surgery.^1^,^2^ Nowadays, antiplatelet and anticoagulants are commonly prescribed for thromboprophylaxis in various conditions.^3^ A wide variety of antithrombotic ranging from antiplatelet like aspirin, clopidogrel, ticagrelor to anticoagulants like warfarin and direct oral anticoagulants have been used for preventing thromboembolism. The increasing burden of such patients has led to a higher number of individuals reporting for emergency or elective surgical procedures under continued antithrombotic therapy.^4^,^5^

While treating patients under antithrombotic, the clinician has to balance the risk of bleeding against the risk of thromboembolism associated with discontinuation of these drugs.^6^ The risk can be altered by the type of surgical procedure. Indeed, bleeding for a simple laceration cannot be compared with intra-operative bleeding associated with gastrointestinal (GI) surgeries. Similarly, a laparoscopic cholecystectomy does not result in the same amount of bleeding as compared to resection for colorectal cancer. Therefore, it is imperative that the management of patients under antithrombotic for different surgical procedures is clearly defined.^7^

Gall bladder disease is one of the most common indication for abdominal surgery.^8^ Around 11% of Chinese population suffers from gallstones.^9^ Cholecystectomy is routinely indicated for managing gallstone disease and due to the high frequency of this surgical procedure, it is imperative to understand if the surgery can be safely performed in patients under antithrombotic medications. Several studies have reported outcomes of cholecystectomy in patients on antithrombotic drugs but with small sample size and varied results.^10^-^12^ Furthermore, no systematic review has attempted to pool evidence. Therefore, we hereby aimed to systematically analyze literature in order to answer the following clinical query: Do patients under antithrombotic medications have a higher risk of bleeding and bleeding-related complications while undergoing cholecystectomy as compared to patients not under any such drugs?

## METHODS

The review was prospectively on PROSPERO (CRD42021283677). The reporting guidelines of the PRISMA statement were also followed.^13^

### Literature search:

Two reviewers electronically searched PubMed, Embase, Science Direct, CENTRAL, and Google Scholar databases from inception up to 20^th^ January 2022. The keywords utilized were “aspirin”, “clopidogrel”, “warfarin”, “direct oral anticoagulants”, “antiplatelet”, “anticoagulants”, “anticoagulation”, “antithrombotic”, and “cholecystectomy” in various combinations (Supplementary Table-I). We then electronically reduplicated the results and screened studies by their titles and abstracts. Articles relevant to the subject of our review were identified and their full texts were extracted. These articles were then examined by two reviewers independently for final inclusion in the review. Any discrepancies in study selection were resolved by consensus. Finally, we also searched the reference list of included studies to look for any other possible inclusions.

### Eligibility criteria:

We included: 1) All types of studies conducted on patients undergoing emergency or elective cholecystectomy using open or laparoscopic techniques. 2) Studies were to compare outcomes of patients under any antithrombotic medication vs a control group of patients not under any such drugs 3) Studies were to report data on any of the following outcomes: intra-operative blood loss, blood transfusions, postoperative bleeding complications, conversion to open surgery (for laparoscopic procedures), and length of hospital stay (LOS).

### Exclusion criteria:

- Studies on a mixed cohort of general surgery patients not reporting separate outcomes for cholecystectomy
- Studies not reporting any relevant outcome
- Non-English language studies 4) Studies reporting duplicate data.

### Data extraction and quality assessment:

Two authors independently extracted the following data: author details, publication year, study type, study database, patient population, sample size, type of antithrombotic, demographic details, surgery type (open or laparoscopic), operating time, the perioperative management protocol, and international normalization ratios (INR).

The methodological quality of studies was assessed using the Newcastle-Ottawa scale (NOS).^14^ It was conducted by two authors independent of each other. Any disagreements were solved by a discussion. Studies were assessed for selection of study population, comparability, and outcomes, with each domain being awarded a maximum of four, two, and three points respectively. The maximum score which can be awarded was nine.

### Statistical analysis:

The meta-analysis was performed using “Review Manager” (RevMan, version 5.3; Nordic Cochrane Centre (Cochrane Collaboration), Copenhagen, Denmark; 2014). Continuous variables were pooled using mean difference (MD) with 95% confidence intervals (CI) while dichotomous data were pooled using odds ratios (OR) with 95% CI. If continuous data was reported by the studies as median and range, we imputed mean and standard deviation values using methods reported by Wan et al.^15^ All meta-analyses were conducted using the random-effects model. Heterogeneity was assessed using the I2 statistic. We did not assess publication bias using funnel plots as <10 studies were available for analysis. A sensitivity analysis was carried out to assess the contribution of each study on the review results. Subgroup analyses were carried out based on the type of cholecystectomy and type of antithrombotic.

## RESULTS

**Fig.1:**
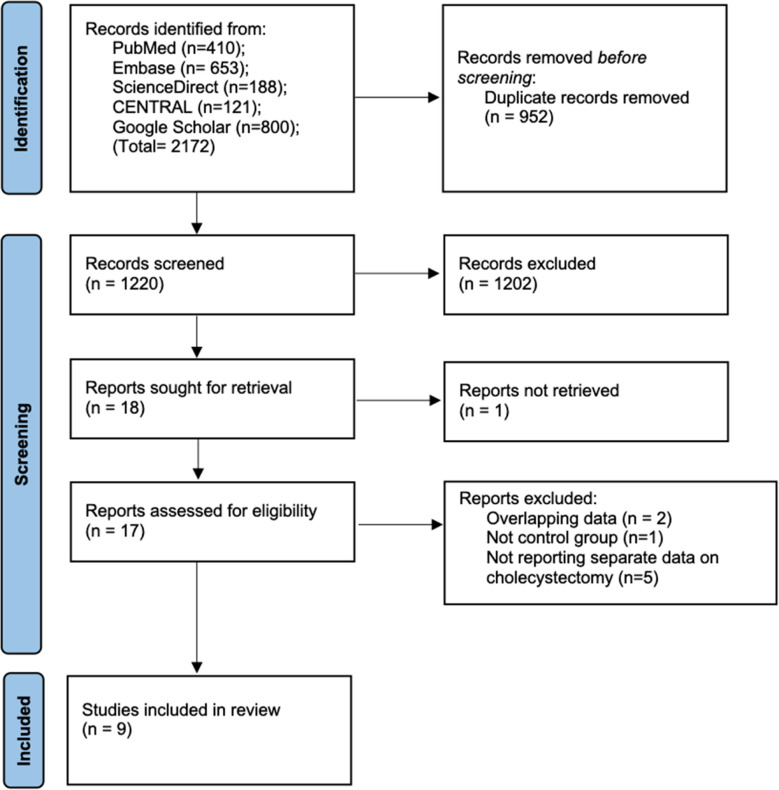
Study flow chart.

The literature search revealed 2172 articles. Fig.1. These were then reduplicated to exclude 952 records. We screened 1220 articles by their titles and abstracts and selected 18 studies for full text analysis. One full text was unavailable. The remaining 17 studies were assessed by the reviewers and eight articles were excluded with reasons. Finally, nine studies were included in the systematic review and meta-analysis.^10^-^12^,^16^-^21^

Four studies were from Japan, two each from the USA and Turkey while one was from Italy. Table-I The two studies from Turkey were from the same institute but had different study periods. Except for two, all studies were retrospective in nature. Two studies included patients only on warfarin while another three included only those on antiplatelet drugs. In the remaining four studies, patients on all types of antithrombotic drugs were included. Five studies were exclusively on laparoscopic cholecystectomies while the remaining included a mix of open and laparoscopic procedures.

Six of the nine studies were on emergency cholecystectomies, two included only planned procedures while one study included a mix of both patients. The perioperative protocol for managing patients under antithrombotic was more or less similar across the included studies. For elective procedures, anticoagulant drugs were stopped and patients were bridged on heparin, and INR was maintained <1.5 during surgery. For emergency cases, anticoagulated patients with INR>1.5 were administered with vitamin K or fresh frozen plasma but patients were taken up for surgery without awaiting normalization of INR. All antiplatelet drugs were continued up to surgery and there was no discontinuation of drug therapy for such patients. The NOS score of the studies ranged from seven to nine.

### Outcomes:

**Fig.2A:**
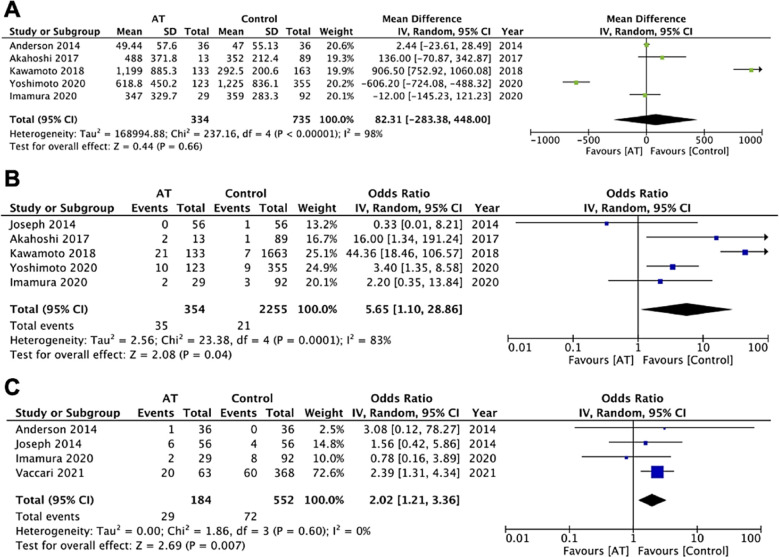
Meta-analysis of intraoperative blood loss in patients undergoing cholecystectomy based on use of antithrombotics (AT). Fig.2B: Meta-analysis of blood transfusion in patients undergoing cholecystectomy based on use of antithrombotics (AT). Fig.2C: Meta-analysis of conversion to open surgery in patients undergoing laparoscopic cholecystectomy based on use of antithrombotics (AT).

Data on intra-operative blood loss was reported by five studies. Overall, the meta-analysis revealed that the use of antithrombotic medications had no statistically significant effect on intra-operative blood loss in patients undergoing cholecystectomy (MD: 82.31 95% CI: -283.38, 448 I2=98% p=0.66). Fig-2A there was no change in the significance of results on the exclusion of any study during sensitivity analysis. The results were statistically non-significant even on subgroup analysis based on the type of cholecystectomy and type of antithrombotic. Table-II

Data on perioperative blood transfusion was also reported by five studies. Meta-analysis indicated that patients under antithrombotic therapy were at an increased incidence of perioperative blood transfusion (OR: 5.65 95% CI: 1.10, 28.86 I2=83% p=0.04). Fig.2B there was no change in the significance of the results on the exclusion of any study. Results of subgroup analysis indicated that patients under antithrombotic drugs did not have an increased incidence of blood transfusion in studies on laparoscopic procedures, however, the results were significant for studies including a mixed cohort of patients. Table-II Similarly, the risk was statistically non-significant for studies only on antiplatelet but significant for other studies.

**Fig.3A:**
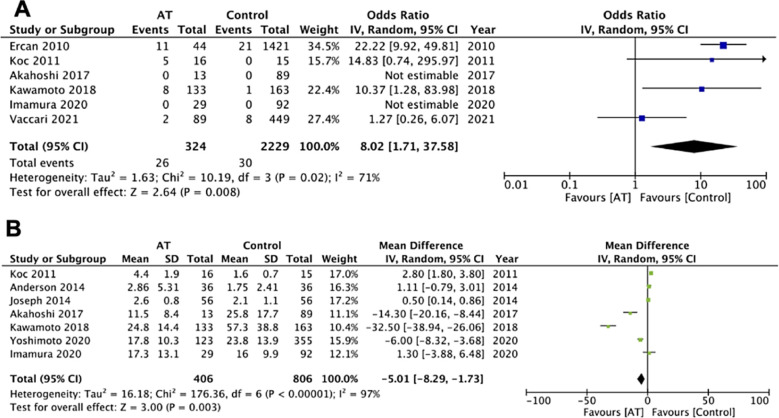
Meta-analysis of bleeding complications in patients undergoing cholecystectomy based on use of antithrombotics (AT) Fig.3B: Meta-analysis of LOS in patients undergoing cholecystectomy based on use of antithrombotics (AT)

Four studies reported data on conversion to open surgery for laparoscopic cases. Pooled analysis indicated that cholecystectomy patients under antithrombotic are at an increased risk of conversion to open surgery (OR: 2.02 95% CI: 1.21, 3.36 I2=0% p=0.007) (Fig.2C). On sensitivity analysis, the exclusion of the study of Vaccari et al^12^ resulted in no significant difference between the two groups (OR: 1.29 95% CI: 0.49, 3.41 I2=0% p=0.61). Furthermore, the incidence of bleeding complications was also found to be significantly increased in patients under antithrombotic therapy (OR: 8.02 95% CI: 1.71, 37.58 I2=71% p=0.008). Fig-3A On exclusion of the study of Ercan et al^21^ (OR: 4.37 95% CI: 0.85, 22.41 I2=44% p=0.08) and Kawamoto et al^17^ (OR: 7.34 95% CI: 0.90, 59.69 I2=80% p=0.06) the results turned non-significant. Subgroup analysis for these outcomes was not conducted due to a limited number of studies and the absence of bleeding complications in some of the included studies.

Seven studies reported data on LOS. Meta-analysis revealed significantly shorter LOS in patients under antithrombotic (MD: -5.01 95% CI: -8.29, -1.73 I2=97% p=0.03). Fig.3B on the exclusion of the study of Kawamoto et al^17^ the results turned non-significant on sensitivity analysis (MD: -1.38 95% CI: -3.76, 1.00 I2=93% p=0.26). Subgroup analysis based on the type of cholecystectomy indicated that LOS was not significantly different between the two groups when only laparoscopic studies were included, however, the results did indicate reduced LOS in studies including a mixed population. On subgroup analysis based on the type of antithrombotic, there was no difference in the LOS in studies on antiplatelet only and studies including all antithrombotic. Table-II

## DISCUSSION

Our review data pooled from nine observational studies to assess if cholecystectomy can be safely performed in patients under antithrombotic. An unforeseen strength of the review was the similar perioperative protocol in included studies for managing patients under antithrombotic. All antiplatelet drugs were continued up to surgery. For anticoagulants, patients were administered vitamin K or fresh frozen plasma if INR was >1.5 whereas for elective procedures bridging therapy with heparin was used. Including data from all possible studies, we noted that intra-operative blood loss was not significantly different in patients under antithrombotic vs controls, however, the incidence of blood transfusions and risk of bleeding complications were significantly higher in patients under antithrombotic. However, one must be cautious in interpreting these results as there was very high heterogeneity in our meta-analysis. For intra-operative blood loss, three studies noted no difference between the two groups, however, there were two outliners with Kawamoto et al^17^ reporting higher blood loss in the antithrombotic group and Yoshimoto et al^11^ reporting opposite results. It must be specified that both these studies did not report blood loss as mean and SD but only as median and range. The mean and SD values were computed for these studies and this may be one reason for the conflicting outcomes. In the study of Yoshimoto et al^11^, the median blood loss in the antithrombotic and control groups was 80 (0-2315) and 10 (0-4880) respectively (p<0.005), with lower median values in the antithrombotic group but a higher upper limit in the control group. Secondly, the incidence of perioperative blood transfusion, which is a surrogate marker of bleeding, was also higher in the antithrombotic group in both the studies of Kawamoto et al^17^ and Yoshimoto et al.^11^ Thus overall, based on the current limited evidence, it seems that cholecystectomy patients under antithrombotic drugs have an increased risk of bleeding and bleeding-related complications.

While interpreting bleeding results, it is important to differentiate between antiplatelet and anticoagulant drugs. Continuation or interruption of antiplatelet in the perioperative period has been a controversial issue and a subject of many randomized controlled trials. In a study of 10,010 patients, the POISE-2 investigators have reported that perioperative aspirin is associated with an increased risk of major bleeding complications in patients undergoing non-cardiac surgery.^22^ However, a meta-analysis has reported that continued antiplatelet therapy during non-cardiac surgery confers only a minimal bleeding risk with no difference in thrombotic complications^5^. Similar results have been reported by studies on various GI surgeries. Takahashi et al^23^ have noted no increase in the risk of bleeding and other complications with continued perioperative use of aspirin in laparoscopic colorectal cancer surgery. Similarly, Monden et al^24^ have shown that discontinuation of aspirin is not needed for patients undergoing liver resections. Ohya et al^4^ in a multicentric study have shown no difference in bleeding with continuation/interruption of aspirin in the perioperative period in colorectal cancer patients. However, this is not the case when GI surgeries are carried out in patients under continued anticoagulant drugs. Harada et al^25^ have reported increased rates of bleeding with continued anticoagulant therapy in colorectal polyp patients undergoing endoscopic snare resection. Similar outcomes have been reported in anticoagulated patients undergoing colorectal endoscopic submucosal dissection.^26^ Sonamura et al^27^ have noted an increased risk of bleeding in gastric cancer surgery patients under warfarin receiving bridging therapy with heparin and those under direct oral anticoagulant drugs. In our review, we attempted a subgroup analysis based on the type of antithrombotic drugs but could include just two studies in the antiplatelet subgroup. While these two studies reported no difference in bleeding-related outcomes, the data is too scarce to derive strong conclusions. Furthermore, we were unable to report separate outcomes for anticoagulants due to limited data from included studies. Future studies on cholecystectomy patients should segregate data on antiplatelet and anticoagulant drugs to provide better evidence.

Another variable influencing bleeding outcomes is the invasiveness of cholecystectomy. In our subgroup analysis, we noted that intra-operative blood loss and incidence of blood transfusion were not significantly different in patients undergoing laparoscopic surgeries. On the other hand, the incidence of blood transfusion was higher in patients on antithrombotic when studies on both open and laparoscopic procedures were included. Indeed, the number of studies in our analysis was not high enough to provide quality evidence. However, our data also suggested that in patients undergoing laparoscopic surgeries the use of antithrombotic is associated with an increased risk of conversion to open surgery. This is indicative of the fact that higher intraoperative bleeding would have necessitated an increased risk of such conversions. Nevertheless, researchers have shown that compared to open surgery, laparoscopic surgeries are associated with reduced bleeding complications. Sulu et al^28^ have shown that the laparoscopic approach was associated with lower thromboembolic and hemorrhagic complications than open surgery in colorectal resection. Similar results have been reported by Nozawa et al.^29^ Further studies comparing outcomes of laparoscopic and open cholecystectomy in patients on antithrombotic are needed to clarify the difference in the risk of bleeding between the two approaches.

In our analysis, we noted that the LOS was lower in patients under antithrombotic as compared to controls. Such results are in direct contrast with the bleeding outcomes noted in our meta-analysis. It is plausible that since patients on antithrombotic had a higher risk of bleeding and bleeding complications these would require longer monitoring in a healthcare setup which would increase LOS. Indeed, our results were significantly influenced by the outliner study of Kawamoto et al^17^, exclusion of which indicated no difference in the LOS between the two groups.

### Limitations:

The limitations of our review need to be specified. Foremost, limited number of studies were available for review. The sample size was also small in many studies. Secondly, the quality of evidence cannot be deemed high due to the observational nature of the studies. Furthermore, the two study groups were not matched for baseline differences in all studies. Lack of propensity score matching could have influenced the study results. Thirdly, most of the studies included a mix of patients with antiplatelet and anticoagulant drugs. This precluded clear evidence on the influence of these two drug types on the study outcomes. The included studies were also heterogeneous in the approach to cholecystectomy and included a mix of open and laparoscopic and elective and emergency procedures.

## CONCLUSION

Current evidence from a limited number of studies indicates that the use of antithrombotic may be associated with an increased risk of bleeding-related complications in patients undergoing cholecystectomies. Antithrombotic use may also increase the risk of conversion to open surgery in patients undergoing laparoscopic cholecystectomies. Further studies with a larger sample size reporting separate data based on the type of cholecystectomies and antithrombotic are needed.

### Authors’ contributions:

**XS** conceived and designed the study.

**XS** **and XC** collected the data and performed the analysis.

**XS** was involved in the writing of the manuscript and is responsible for the integrity of the study.

All authors have read and approved the final manuscript.
